# Biosynthesis of bromoform by *Curvularia* fungi provides a natural pathway to mitigate enteric methane emissions from ruminants

**DOI:** 10.1016/j.btre.2025.e00876

**Published:** 2025-01-14

**Authors:** Thomas Loan, Avinash Karpe, Saeid Babaei, Stuart Denman, Chunghong Chen, Matthias Joust, Kristy Lam, Dawar Hussain, Sapna Pillai Vibhakaran, Matthew Callaghan, Abed Chaudhury, Karen Paco, Nigel Tomkins, Tristan Yang, Stephanie Payne, Michael Ayliffe, Ming Luo

**Affiliations:** a CSIRO Agriculture &Food, Box 1700, Clunies Ross Street, Canberra, Australia; b CSIRO Agriculture &Food, St. Lucia, Brisbane, Australia; c LoamBio Pty Ltd, Charles Sturt University, Orange, NSW, Australia; d School of Agriculture and Environment, University of Western Australia, Crawley, WA, Australia

**Keywords:** Agriculture, Ruminant livestock, Methane emissions, Bromoform, Curvularia

## Abstract

The ruminant livestock industry is the largest agricultural emissions source of anthropogenic greenhouse gases, primarily from enteric methane. Several technologies, both natural and synthetic are being investigated to mitigate enteric methane emissions. Macroalgae derived feed ingredients that contain bromoform, a recognised inhibitor of methanogenesis, are an effective natural approach for methane mitigation. However, producing sufficient biomass economically to satisfy the livestock industry at a global scale is challenging. Here we demonstrate that a *Curvularia* soil fungi isolate can be cultured to produce bromoform and subsequently inhibit methanogenesis in pure cultures of *Methanobrevibacter smithii* and in mixed cultures of ovine rumen fluid. This highly culturable fungal species produces no known toxins and creates an exciting new, scalable and natural alternative for reducing ruminant livestock emissions.

## Highlights

• Methane emissions from ruminant livestock are a major greenhouse gas source.
• Bromoform feed additives can reduce livestock methane by >90 %.
• *Curvularia* soil fungi produce bromoform and prevent rumen methane emission.
• These natural, culturable fungi offer a unique climate change strategy.

## 1 Introduction

The world's livestock industry produces 14 % of global anthropogenic greenhouse gas emissions, mostly as enteric methane [1]. Methane is produced as a by-product of anaerobic fermentation by methanogenic archaea present in the rumen of livestock [2]. Enteric methane production is also reported to cause a 2–15 % loss of dietary gross energy intake [3,4]. Mitigation of enteric methane emissions would be highly advantageous, both in helping to reduce greenhouse gas emissions from the agricultural sector and improving productivity efficiencies, particularly as the global meat and milk industry is predicted to increase from 2010 levels by >50 % in 2050 [1].

Trihalogenated compounds like chloroform and bromoform significantly reduce methanogenesis by inhibition of cobamide-dependent methyl-transfer reactions [5,6]. These compounds suppress bacterial methanogenesis by reacting with the B12 cofactor of the methyltransferase complex responsible for the formation of methyl co-enzyme M in the penultimate step of methanogenesis [2,7].

Halogenated compounds (*i.e.*, containing Br, Cl or I) are produced biologically by vanadate-dependent haloperoxidase enzymes (VHPOs) which oxidise halogens in the presence of H_2_O_2_ to produce hypohalous acids. These acids react with nucleophilic receptors to form halogenated compounds [8]. The VHPO enzymes include chloro-, bromo- and iodoperoxidases. Chloroperoxidases utilise all three halogens, bromoperoxidases generate brominated and iodinated compounds and iodoperoxidases are limited to iodination reactions [9]. Many algae use bromoperoxidases to produce bromoform (CHBr_3_) which diffuses or is transported out of cells to act as a biofumigant that prevents surface fouling by other organisms [10].

*Asparagopsis taxiformis and A. armata*, marine macroalgae, accumulate high levels of bromoform by storing it in specialised gland cells [10]. When included in ruminant diets *in vivo* or *in vitro* as a feed additive these algae significantly suppress enteric methanogenesis [[11], [12], [13], [14]]. Consequently, *Asparagopsis* derived feed additives for ruminants are being developed, however, it is improbable that sufficient algal biomass can be produced to meet abatement targets in global livestock production systems. Currently the global seaweed industry produces 3–6 million metric tonnes (MMT) dry weight of product for human and animal consumption and industrial use [15]. It is calculated that 3.5 MMT of dried *Asparagopsis* seaweed, over half the world's current total seaweed production, would be required annually to feed the America herd of 93 million cattle. Supplying the entire global herd of 1.4 billion cattle is therefore unfeasible [15]. Other concerns for a ruminant seaweed industry include product variability, the cost of production (*Asparagopsis* takes 10 −11 months to grow and is estimated to be 3–4 times more expensive than synthetic alternatives) and the algae's invasive potential in non-native habitats.

New sources of bromoform containing feed ingredients are required to support international mitigation targets. We have therefore investigated microbial fermentation strategies as alternative natural sources of bromoform. Microbial fermentation has significant advantages over aquaculture in that it is an economical, highly scalable and well-established food additive production process. It is environmentally contained and utilises rapidly growing micro-organisms, with cheap substrate inputs and can be readily located close to market sites thereby minimising transport and supply chain emissions.

Bacteria, fungi and lichens also use VHPO enzymes to synthesize halogenated compounds [16]. Screening of dematiaceous hyphomycete fungal species previously identified chloroperoxidase activity ranging from high to low in 83 of 112 species/isolates tested [17]. The highest VHPO expressing fungi included *Alternaria, Curvularia, Drechslera, Ulocladium* and *Botrytis* species [17]. Partially purified chloroperoxidase extracts from *Curvularia inaequalis* produced a range of halogenated compounds including, 3-chloro-2-bromo-1-propanol; 3-bromo-2-chloro-1-propanol; 2,3-dichloro-1-propanol; 2,3-dibromo-1-propanediol; and chloro and bromo propanediols, depending upon the substrates available [17].

The VHPO enzyme of the saprophytic fungus *C. inaequalis* [[18], [19], [20], [21]] has both chloroperoxidase and bromoperoxidase activity [19]. This secreted peroxidase is a 609 amino acid protein encoded by a single *VHPO* gene in a C. *inaequalis* isolate examined [8]. In saprophytic fungi like *C. inaequalis*, these chloroperoxidases are believed to facilitate the oxidative degradation of plant cell walls [8,[21], [22], [23]].

The ability of VHPO expressing fungal species to provide alternative, natural sources of bromoform at commercially viable concentrations for enteric methane mitigation has not been shown. Here we demonstrate the potential of a *Curvularia* isolate to bio-manufacture a novel bromoform source for the mitigation of enteric methane.

## 2 Materials and methods

### 2.1 Fungal cultures

Ninety-nine fungal cultures, listed in Table S1, were provided by LoamBio Pty. Ltd. (Orange, Australia) and grown on 10 cm potato dextrose agar (PDA) plates (20 g/L glucose, 4 g/L potato extract and 20 g/L agar) at 28°C until sporulation. Plates were stored at 4°C. For subsequent inoculations either spores were harvested by flooding plates with water and the spore suspension, or small pieces of fungal infected PDA media used directly for inoculation. Fifty four of these 99 isolates (Supplementary Table S1, highlighted in grey shading) were tested for VHPO activity and 65 of these isolates were genome sequenced (Supplemental Table S1, column E), as described below.

### 2.2 VHPO expression of *Curvularia* and related fungal isolates using the phenol/red assay

Fifteen mL of liquid minimal media [0.5 % yeast extract and 0.2 % mineral solution (4 mM K_2_HPO_4_, 2.5 mM CuSO_4_, 2.75 mM FeSO_4_, 4 mM MnCl_2_, 5 mM ZnSO_4_] + 0.5 g/L glucose were inoculated with 100 µL of fungal spore suspension and incubated at 28°C for 14 d with shaking at 100 r.p.m. Both culture media supernatant and fungal mycelial extracts were quantified for VHPO activity using a phenol red assay [24]. In brief, 100 µL of culture supernatant was added to 100 µL of phenol red solution containing 3-morpholinopropane-1-sulfonic acid (MOPS, 100 mM, pH 7.0), KBr (50 mM), phenol red (0.45 mM), Na_3_VO_4_ (1 mM). The reaction was initiated with the addition of 20 mM H_2_O_2_ and incubated at room temperature for 2 h and 24 h, respectively. A similar assay was also conducted on fungal mycelial biomass whereby hyphae were isolated from cultures as a single mat and rinsed in sterile water to remove any remaining culture solution. Hyphal samples were then freeze dried for 24 h under vacuum (200 mTor, -40°C) and added to 2 mL tubes along with a 3 mm ball bearing. Tissue was then homogenised in a TissueLyser II (Invitrogen, USA) at 30 Hz until homogenous and resuspend in 1 mL of water. This suspension was centrifuged (14,000 x g, 2 min) and 100 µL of supernatant was added to the phenol red assay as described above. In both assays VHPO activity was detected by the conversion of phenol red to bromophenol blue and this chromogenic product quantified by absorbance at OD_590nm_ in 96 well flat bottom plates using a FlUOstar Omega microplate reader (BMG Labtech, Germany).

### 2.3 VHPO enzyme kinetics

Fungal cultures were grown as described and 100 µL sample*s* extracted at days 10–14 inclusive. At each time point 100 µL of culture supernatant was added to 100 µl of phenol red assay as described. Changes in absorbance at OD_590nm_ of each reaction were measured every minute over 2 h and VHPO activity calculated as change in OD_590nm_ absorbance per second.

### 2.4 Genomic sequencing of fungal isolates

Fungal isolates were grown in 25 mL of minimal media for 14 d at 28°C with shaking, after which time mycelia were harvested by centrifugation (5000 x g, 10 min). Samples were sequenced at the Australian Genome Research Facility using the Illumina NextSeq sequencing platform. Genome sequences were then produced by *de novo* assembly using the SPAdes program (version 3.15.2) in “isolate” mode. *VHPO* gene homologues were identified in these fungal genomes by homology searches using the cloned *C. inaequalis* gene isolated from Central Bureau voor Schimmelcultures strain 102.42 (GenBank X85369) as a query template [8].

### 2.5 Protein structural predictions and similarity

*VHPO* DNA sequences were translated and protein structures modelled using Alphafold [25]. VHPO proteins were compared to each other and the published *C. inaequalis* protein sequence (GenBank: CAA59686.1) by multiple alignment using Clustal Omega and default parameters [26].

### 2.6 Synthesis of bromoform by fungal isolate 4388

To test for inhibition of methanogenesis by *M. smithii*, twenty cultures of fungal isolate 4388 were each grown in 15 mL of LMM + 0.5 g/L glucose at 28 °C for two weeks, and then pooled into two samples of equal weight (105 g each). Each pool was supplemented with 0.1 mol/L N-(2-Hydroxyethyl) piperazine-N′- (2-ethanesulfonic acid) (HEPES, pH 7.6), 0.75 mmol/L Na_3_VO_4_, 2 mmol/L acetyl acetone and 90 mmol/L H_2_O_2_. In one sample, 30 mmol/L KBr was included and omitted in the other. Both samples were stirred (50 rpm) at room temperature for 6 d whereupon a further 90 mmol/L H_2_O_2_ was added. Following a further day of incubation (total 7 d at room temperature) supernatant was separated from fungal mycelia by centrifugation (5000 x g, 10 min) and used for *M. smithii* growth inhibition experiments.

For optimal bromoform synthesis cultures of fungal isolate 4388 were grown in LMM + 8 g/L glucose with 0.4 g/L agar for 14 d and then supplemented with 0.05 mol/L N-(2-Hydroxyethyl) piperazine-N′- (2-ethanesulfonic acid) (HEPES, pH 7.4) and 100 µmol/L Na_3_VO_4_. For a further four consecutive days 0.02 mol L^-1^ d^-1^ KBr, 0.02 mol L^-1^ d^-1^ 5,5-dimethyl-1,3-cyclohexadione and 0.1 mol L^-1^ d^-1^ H_2_O_2_ were added to each sample. To compare the effects of fungal biomass or culture supernatant on rumen fermentation, fungal cultures which contained up to 10 mM CHBr_3_, were either homogenised on ice in 10 mL samples by sonication with a Q500 Sonicator (Q sonica, USA) at 30 % amplitude, 50 % pulse length for 2 min (fungal biomass samples) or centrifuged and supernatant samples collected. Sonication was necessary for biomass samples to enable aliquoting of a uniform homogenate to accurately replicate experimental samples.

### 2.7 Detection of bromoform

With the exception of the *in vitro* experiment using ovine rumen fluid, SPME-GCMS analysis was used to determine the concentration of bromoform [27]. Briefly either a 7890A series gas chromatograph with a 5975C inert XL MSD detector (Agilent, USA) and an MPS2 autosampler (Gerstal, Germany) or an 8890 series gas chromatograph with a 7250Q/TOF mass selective detector (Agilent, USA) and MPS3 2XL autosampler (Gerstal, Germany) was used, each with a VF-5 ms column (30 m x 0.25 mm x 0.25 μm) and 10 m EZ -guard column (Agilent, USA). Culture headspaces were sampled by adsorption on divynlbenzene /carboxen /polydimethylsiloxane13 fibre (Supleco, USA), before desorption at 250°C for 5 min with a 1.0 mm straight, no-wool liner in split less injection mode. The carrier gas was ultra-high purity He (BOC Ltd, Australia) at a flow rate of 1 mL/min. The aux transfer line was held at 320°C, the ion source at 250°C and the quadrupole at 150°C. Electron impact ionisation energy was 70 eV, with M/S scan from m/z 40-700. The oven was held at 40°C for 2 min, ramped up to 150°C at 5°C/min, held for 2 min, then ramped to 320°C at 15°C/min and held for 1 min. The fiber was reconditioned at 250°C for 10 min after injection to prevent carryover. Bromoform was identified by comparison of retention time with an authentic standard (Merck, USA) and by mass spectral matching using the NIST/EPA/NIH mass spectral library (version 2014). Bromoform was quantified by comparison of the signal corresponding to the molecular ion (m/z = 252 ± 0.5) with a standard curve.

For quantification of bromoform in fungal extracts used for *in vitro* testing in ovine rumen fluid a Shimadzu GC-2030 gas chromatograph was used fitted with a SH-I-5Sil column (30 m x 0.25 mm x 0.25 µm), a HS-20NX headspace sampler module and a GCMS-QP2020NX single quadrupole mass spectrometer. Sample line and transfer line temperatures of 150 °C and 150 °C, respectively, were used. Oven temperature was set to 50 °C and ramped to 150 °C at 35 °C/min, then to 200 °C at 50 °C/min and finally to 250 °C at 70 °C/min. A 50-ratio split injection was used with a gas pressure of 147.0 kPa and total column flow of 54.0 mL/min. For the mass spectrometer, ion source and interface temperatures of 230 and 220 °C, respectively, were used. The spectrometer was tuned using perfluorotributylamine to maximize the m/z 502 peak.

### 2.8 RNAseq analysis

Triplicate cultures of *Curvularia* isolate 4388 were grown for 14 d in LMM [0.5 % yeast extract and 0.2 % mineral solution (4 mM K_2_HPO_4_, 2.5 mM CuSO_4_, 2.75 mM FeSO_4_, 4 mM MnCl_2_, 5 mM ZnSO_4_)] + 8 g/L glucose with shaking at 180 r.p.m. Culture aliquots were taken at days 3 to 14 inclusive and RNA extracted from fungal mycelia by freezing in liquid nitrogen and grinding to a powder with a pestle and mortar before extracting RNA using an RNeasy mini kit (Invitrogen, USA). RNA samples were paired-end Illumina sequenced with six gigabase sequencing depth for each sample by NovageneAIT Genomics Singapore Pty. Ltd. Sequence data was manipulated using FastQC with default parameters and sequences trimmed using Trimomatic to remove adapter sequences. Reads were mapped to *Curvularia* coding sequences using htseq and the number of individual Illumina reads that mapped to each full-length coding sequence determined and averaged for three replicates. Expression of 11,200 different genes was detected. Differential gene expression was identified by comparing the transcript abundance of each gene between samples from sequential days *i.e.* day 3 verses 4, 4 *versus* 5, 5 *versus* 6 etc. Transcripts with a probability difference <0.05 were selected as being differentially expressed in each comparison. A total of 635 genes were identified as differentially expressed in at least one timepoint by this analysis. An expression heat map was produced using Hierarchical Cluster Analysis to visualise expression differences across samples (Supplementary Fig. S4).

### 2.9 Metabolomics analysis

Biomass and supernatant samples from triplicated cultures, as described above and used for RNAseq, were analysed by the Australian Wine Research Institute (AWRI)/Metabolomics Australia for polar metabolites using hydrophilic interaction chromatography (HILIC) LC-MS. Supernatant samples (2 mL) were diluted to 10 mL with milliQ water and metabolites concentrated by solid phase extraction (SPE) using Strata-X polymeric SPE cartridges (Phenomenex, USA). Eluted fractions were dried under nitrogen at 28°C and resuspended in a mixture of 75 μL solvent A (0.1 % formic acid, 0.5 % methanol in MilliQ water) and 25 μL solvent B (0.1 % formic acid, 2 % MilliQ water and 40 % acetonitrile in water) before analysis. For biomass samples, 1.8 mL of 75 % methanol plus 25 % chloroform was added to 50 mg of freeze-dried fungal mycelia and extracted for 15 min on an orbital shaker. Samples were centrifuged at 14,500 rpm for 14 min, and a second extraction undertaken on the pellet with 600 μL of the methanol/chloroform mixture. The two extracts were pooled, and 1 mL of ice-cold water added before centrifugation at 14,500 rpm for 15 min. The supernatant was then dried under nitrogen and resuspended in solvents A and B. A Kinetex F5 column (150 mm x 2.1 mm internal diameter; 2.6 μm particle size, Phenomenex) at 30°C was used for metabolite separation using a flow rate of 0.4 mL/min with linear elution gradients of 0 to 1 % solvent B over 6.25 min, to 7.5% solvent B over 13.75 min, to 60 % solvent B over 10 min and 90 % solvent B over 3 min. The gradient was held at 90 % solvent B before washing and re-equilibrating the column. Samples were injected for data collection in MS1 mode (4 μL) and MS2 mode (8 μL). Mass spectral data were collected with a OrbitrapTribrid IDX MS (Thermoscientific, USA), equipped with a heated electrospray ionisation source, under the following conditions: spray voltage 3350 V, sheath gas 25, arbitrary units (au), sweep gas 1 au, auxiliary gas 10 au, ion transfer temperature 275°C, and vaporizer temperature 300°C. Compounds were identified by matching MS2 spectra to both an AWRI compound reference library and the mzCloud online library mzVault. Molecules that could not be identified were assigned a molecular formula. A heatmap showing compound accumulation across the culture time courses was constructed using Hierarchical Cluster (Fig. 4b; Supplementary Fig. S5). Biochemical pathway analysis was undertaken using Metaboanalyst 6.0 [28] and Paintomics 4.0 [29] to integrate transcriptomic and metabolomics data.

### 2.10 *In vitro* inhibition of methanogenesis and growth of *M. smithii* by bromoform containing culture extracts from fungal isolate 4388

Replicated tubes containing 9 mL of BN medium were inoculated with 250 μL of an overnight culture of actively growing *M. smithii* cultures. Culture filtrates from +KBr and -KBr cultures of fungal isolate 4388 were also immediately added, and samples gassed to 120 kPa with H_2_ and grown in the dark at 39°C with shaking (50 rpm) for 16 h. Gas pressures were recorded at 16 h and 3 mL of head space was analysed by GC-MS to measure methane concentration.

### 2.11 Inhibition of methanogenesis of an advanced *M. smithii* culture by a bromoform producing culture of fungal isolate 4388

*M. smithii* cultures were grown for 16 h in 9 mL of BN media and then 1 mL of fungal isolate 4388 culture supernatants added that were derived from either minimal media or minimal media + 30 mM KBr culture supernatants. As a control, a known methanogenesis inhibitor, 2 µM chloroform, was added to some bacterial cultures. Reactions were triplicated and cultures gassed to a pressure of 120 kPa with H_2_ and grown in the dark at 39°C with shaking (50 rpm) for 16 h. Gas pressures were recorded at 16 h prior to addition of treatments and then at 22 and 40 h (6 and 24 h after treatment) after the addition of culture supernatants. Head space (3 mL) was removed and analysed by GC-MS to determine methane concentration.

### 2.12 Inhibition of methanogenesis *in vitro* by supernatant and homogenate from a bromoform producing culture of fungal isolate 4388

An *in vitro* assay [30], was used to quantify the inhibition of methanogenesis using extracts of fungal isolate 4388. The assays were 24 h batch cultures using pooled rumen fluid collected from three rumen fistulated donor wethers. The animals were acclimatised to animal house conditions and fed a diet of oaten chaff, EasyFibre pellets (Milne feeds, Welshpool WA), lupins and a vitamin mineral premix (Topstock feeds, Bindoon WA) for two weeks prior to rumen fluid sampling. Animals were fed daily at approximately 0800 with additional oaten hay provided in the afternoon. Animals were managed according to protocol 2021/ET001086 approved by the University of Western Australia Animal Ethics Committee. Rumen fluid was collected into pre-warmed thermos flasks and transported immediately to the laboratory. Once in the laboratory the rumen fluid was strained to remove solid particles and then moved into an anaerobic chamber. Time from collection to transfer into anaerobic conditions was < 40 min.

Extracts from bromoform producing cultures of fungal isolate 4388 and control cultures (grown in the absence of KBr) were tested for inhibition of methanogenesis in the *in vitro* assay. The fungal extracts from +KBr cultures contained 0.93 mM and 0.29 mM of bromoform, in culture homogenate and supernatant, respectively. Supernatant and homogenate material from the cultures of fungal isolate 4388 was stored in 20 mL crimped glass vials with minimal headspace at 4 °C until use.

Samples of +KBr homogenate and supernatant were added to the *in vitro* fermentation assay to produce bromoform concentrations ranging from 1 to 10 µM. Briefly, 100 mg substrate (oaten chaff, ground to pass a 1 mm screen) was added to 30 mL vials one day prior to the assay and left in an anaerobic chamber to remove the oxygen. The following day, the appropriate volume of either homogenate or supernatant and 10 mL of rumen fluid inoculum (rumen fluid and buffer [31] in a 1:1.5 (v/v) ratio) was added to each vial and incubated in a shaking incubator at 39 °C for 24 h. Amylene-stabilised bromoform (Sigma Aldrich Cat. No. 36972) was included at the same concentration as +KBr fungal extracts. A substrate only treatment was included as a control. Both homogenate and supernatant from control cultures of fungal isolate 4388 grown in the absence of KBr were added at volumes equivalent to the 10 µM bromoform treatments of +KBr homogenate and supernatant. All treatments were performed in triplicate.

Total gas production, as an indicator of fermentation, was measured using a pressure transducer [30], and subsamples of the head space collected for methane quantification by gas chromatography. Methane yield was calculated as mL of methane per gram of dry matter incubated. Samples of the fermentation fluid were collected for VFA analysis using gas chromatography with an external standard Supelco Volatile Free Acid Mix (Sigma-Aldrich Australia, Cat No. CRM46975,) and an internal standard of 0.9 mM 3-methyl valeric acid in 2.24 w/v% phosphoric acid (Sigma-Aldrich Australia, Cat No. 222453 & 30417). The concentration of NH_3_ in the fermentation fluid was measured spectrophotometrically [32].

## 3 Results

### 3.1 Differential VHPO expression amongst soil fungal isolates

**Fig. 1:**
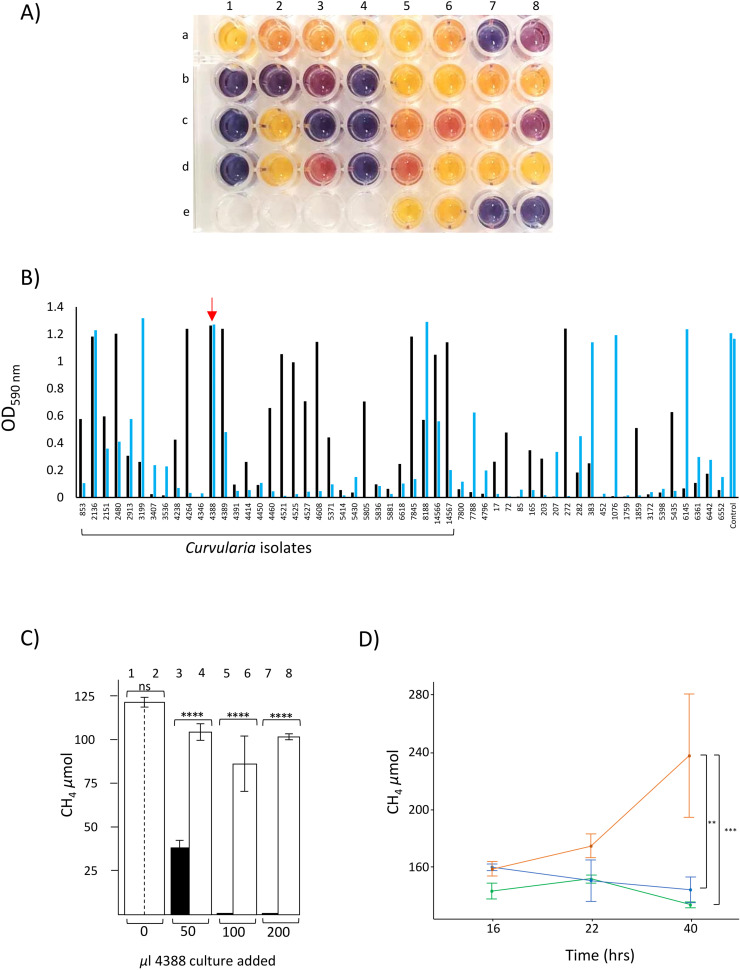
Comparison of VHPO activity in selected fungal isolates. (A) Example of a phenol-red assay plate used to measure VHPO activity in supernatant samples from cultures of *Curvularia* and other fungal genera after 14 d growth. Wells E1–4 were blank while E5 contained buffer only. Well C3 shows VHPO activity from culture supernatant of fungal isolate 4388. Reactions were incubated for 24 h at 28°C. (B) VHPO activity measured by phenol red assay for a selection of fungal isolates (Supplementary Table S1). VHPO activities from culture supernatants are shown as blue columns while VHPO activities of mycelial lysates are shown as black columns. Fungal cultures were grown for approximately 14 d and then VHPO assays undertaken and incubated for 24 h. Data from *Curvularia* isolates are bracketed on the X-axis with fungal isolate 4388 values highlighted with a red arrow. The control (duplicated last sample) was 1 unit of purified bromoperoxidase enzyme from *Corallina officinalis* in media. (C) Methane production by *M. smithii* after addition of 0, 50, 100 and 200 µL of 4388 culture supernatants. Values shown in columns 2, 4, 6 and 8 were from *M. smithii* cultures treated with supernatants from fungal isolate 4388 grown in the absence KBr (bromoform negative) while remaining column values (black) show data from *M. smithii* treated with supernatant from 4388 cultures supplemented with 30 mM KBr (bromoform positive). Each data point is the mean of three reactions. Statistical significance is indicated above compared data pairs (ANOVA, *F* (3,16) = 25.75, *p* = <0.0001, hg2 = 0.83).(D) Inhibition of methanogenesis in advanced *M. smithii* bacteria cultures. Bacterial cultures were grown for 16 h to an advanced stage and then 1 mL of supernatants from fungal isolate 4388 cultures grown in either the presence (green line) or absence (orange line) of 30 mM KBr added. As a control 2 µM of chloroform was added to bacterial cultures as a known methanogenesis inhibitor (blue line). Methane concentrations were measured over a 40 h time course using three biological replicates. Statistically significantly different methane emission curves over time are bracketed (ANOVA, *F* (4,18) = 3.15, *p* = 0.04, hg^2^ = 0.41).

Fifty-four fungal isolates, primarily from the genus *Curvularia* but including *Penicillium, Fusarium, Alternaria* and *Aspergillus* and two unidentified species (Supplementary Table S1, highlighted in grey shading), were tested for VHPO activity. Fungal isolates were grown in liquid minimal media (LMM) [0.5 % yeast extract and 0.2 % mineral solution (4 mM K_2_HPO_4_, 2.5 mM CuSO_4_, 2.75 mM FeSO_4_, 4 mM MnCl_2_, 5 mM ZnSO_4_] + 0.5 g/L glucose for 14 d at 28°C. VHPO enzyme activity was then measured in culture supernatant and in homogenised, freeze-dried mycelial samples by the conversion of phenol red to bromophenol blue and quantified spectrophotometrically at OD_590__nm_ (Fig. 1a) [24]. In this preliminary screen, variable VHPO activities were detected with some *Curvularia* isolates showing high enzyme activities in both supernatant and mycelia samples (Fig. 1b).

### 3.2 Copy number variation the *VHPO* gene

To investigate VHPO genomic architecture, 65 fungal genomes were sequenced including 20 of the isolates tested for VHPO activity above. VHPO copy number variation was found with up to three gene copies present (Supplementary Table S1). Three protein isoforms were also identified: Group I (GI) proteins around 609 amino acids and Group II (GII) and Group III (GIII) proteins approximately 410 and 200 amino acids, respectively (Supplementary Fig. S1). Whether these smaller isoforms retain VHPO function is unknown. Polymorphisms were also apparent with G1 amino acid identities ranging from 48.96−100 % (Supplementary Fig. S1). No relationship between enzyme activity and VHPO copy number or the presence of GII or GIII proteins was observed. Fungal isolates 4388, 2436, and 2780 contained a single GI VHPO gene (Supplementary Table S1) and had high enzyme activity (Fig. 1b). Fungal isolates 5096 and 8100 with two and three VHPO copies, respectively (Supplementary Table S1), showed low VHPO activity (Fig. 1b).

### 3.3 Bromoform produced by cultures of fungal isolate 4388 inhibits *Methanobrevibacter smithii* methanogenesis

*Curvularia* isolate 4388, possibility a *C. clavata* species (Supplementary Fig. S1), was further evaluated given its high VHPO activity (Fig. 1b) and simple genetic architecture, encoding a single GI protein 96 % identical to the published *C. inaequalis* VHPO protein sequence (Supplementary Fig. S2). This isolate was tested for its ability to produce bromoform and inhibit methanogenesis of *Methanobrevibacter smithii,* a readily culturable autotrophic methanogen which inhabits both the rumen and the intestinal tract of humans. Twenty cultures of fungal isolate 4388 were grown in LMM + 0.5 g/L glucose for two weeks. In half the cultures 30 mM KBr was added to enable bromoform production after addition of 100 mM H_2_O_2_ and 2 mM acetyl acetone [27,33]. Bromoform (>10 μM) was detected by SPME-GCMS in +KBr samples and only trace amounts detected in -KBr samples. Aliquots (0, 50, 100, 200 μL) from pooled culture supernatants were added to BN media (9 mL) inoculated with 250 μL of an overnight *M. smithi* culture. After 16 h, substantial methane was detected in head-space samples of control *M. smithii* cultures (Fig. 1c, columns 1, 2.). Addition of supernatant from -KBr treated cultures of fungal isolate 4388 modestly reduced methane production, possibly due to co-addition of oxygen to these anaerobic cultures (Fig. 1c, columns 4, 6, 8). In contrast, methane production was greatly suppressed, in a dose dependent fashion, by supernatant from bromoform producing +KBr cultures (Fig. 1c columns 3, 5, 7).

A second experiment was undertaken that more closely mimics the populous microbial rumen environment. Supernatants (1000 µl) from + and - KBr cultures of fungal isolate 4388 used above were added to 9 mL *M. smithii* cultures that had been actively growing for 16 h. The +KBr culture supernatants inhibited methanogenesis from these dense, pre-established methanogen cultures with similar efficacy to 2 µM chloroform (Fig. 1d). In contrast, supernatants from -KBr cultures of fungal isolate 4388 did not inhibit methanogenesis (Fig. 1d).

### 3.4 Optimisation of fungal isolate 4388 VHPO activity

**Fig. 2:**
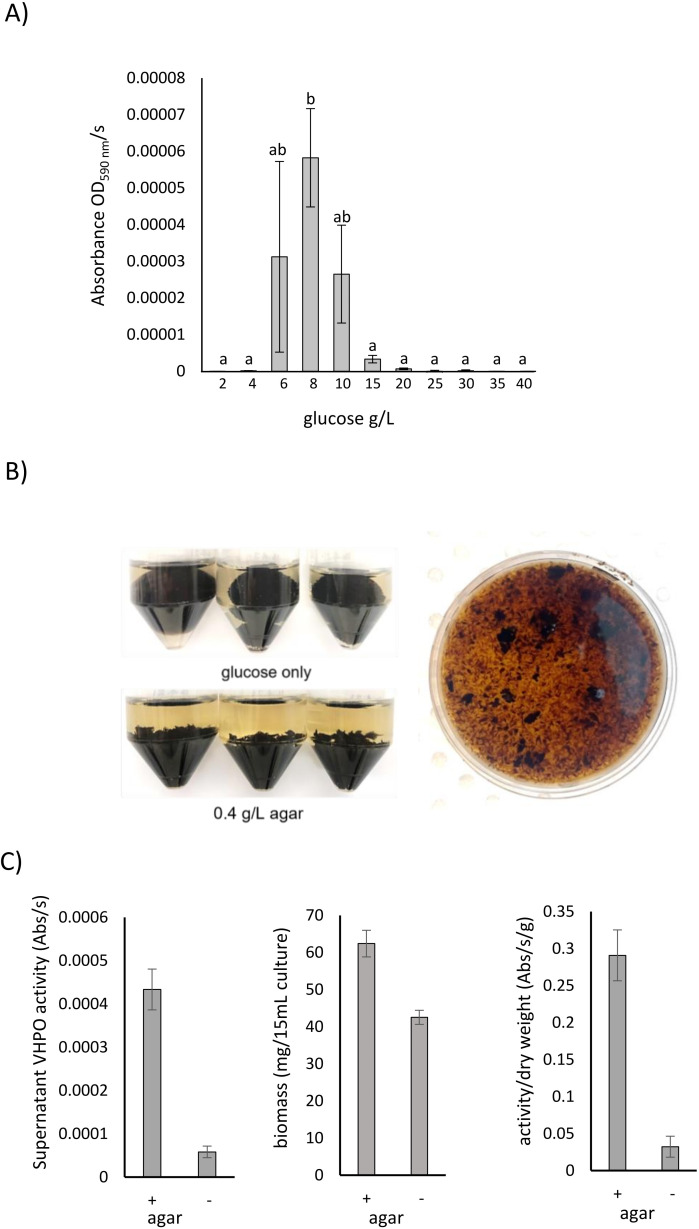
Optimisation of VHPO expression in cultures of fungal isolate 4388. (A) VHPO expression of cultures of fungal isolate 4388 grown in LMM with increasing glucose concentrations. Triplicate cultures were grown with glucose concentrations indicated on the X-axis for 12 d at 28°C with shaking. VHPO enzyme activities were determined from 100 µL aliquots of culture supernatant using the phenol red assay. Columns with common letters are not significantly different (ANOVA *p* < 0.05 was considered significant). (B) Agarose addition to media during culture of fungal isolate 4388 alters fungal morphology. Shown at top left are samples grown in LMM + 8 g/L glucose while the samples below were grown in the same media with the addition of 0.4 g/L agar. Both samples were grown at 28°C for 14 d with orbital shaking (200 rpm). One of the lower tubes was poured into the petri dish shown at right to further show the contents. (C) Agar (0.4 g/L) increases VHPO expression and biomass in cultures of fungal isolate 4388 grown for 14 d at 28 °C. Graphs show VHPO activity present in 100 µL supernatant aliquots (left), fungal mycelial biomass present in 15 mL of culture (centre) and VHPO activity per gram of biomass dry weight (right). Data points are derived from a minimum of three replicate cultures grown in LMM + 8 g/L glucose, with or without the addition of 0.4 g/L agar. Data pairs on each graph are significantly different (T-test, *p* < 0.05).

Media manipulation was undertaken to optimize and increase VHPO enzyme expression of cultures of fungal isolate 4388. LMM with different glucose concentrations or carbon sources or nutrient rich potato dextrose agar were compared, along with different growth regimes. VHPO activity was only detected in LMM + glucose cultures with glucose concentrations significantly affecting enzyme activity (Supplementary Fig. S3). Optimal VHPO activity was obtained when fungal isolate 4388 was grown in LMM + 8 g/L of glucose (Fig. 2a, *P* < 0.05). Increasing glucose concentrations above 8 g/L inhibited VHPO expression (Fig. 2a, Supplementary Fig. S3) suggesting carbon catabolite repression of this gene [34,35]. The addition of 0.4 g/L agar to cultures promoted growth of many smaller foci rather than large mycelial clumps (Fig. 2b) which increased dry weight biomass by 40 % (Fig. 2c; *p* < 0.001) and VHPO activity by 10-fold (Fig. 2c). The combination of these optimisation steps increased VHPO activity in cultures of fungal isolate 4388 by >100-fold (Supplementary Fig. S3).

**Fig. 3:**
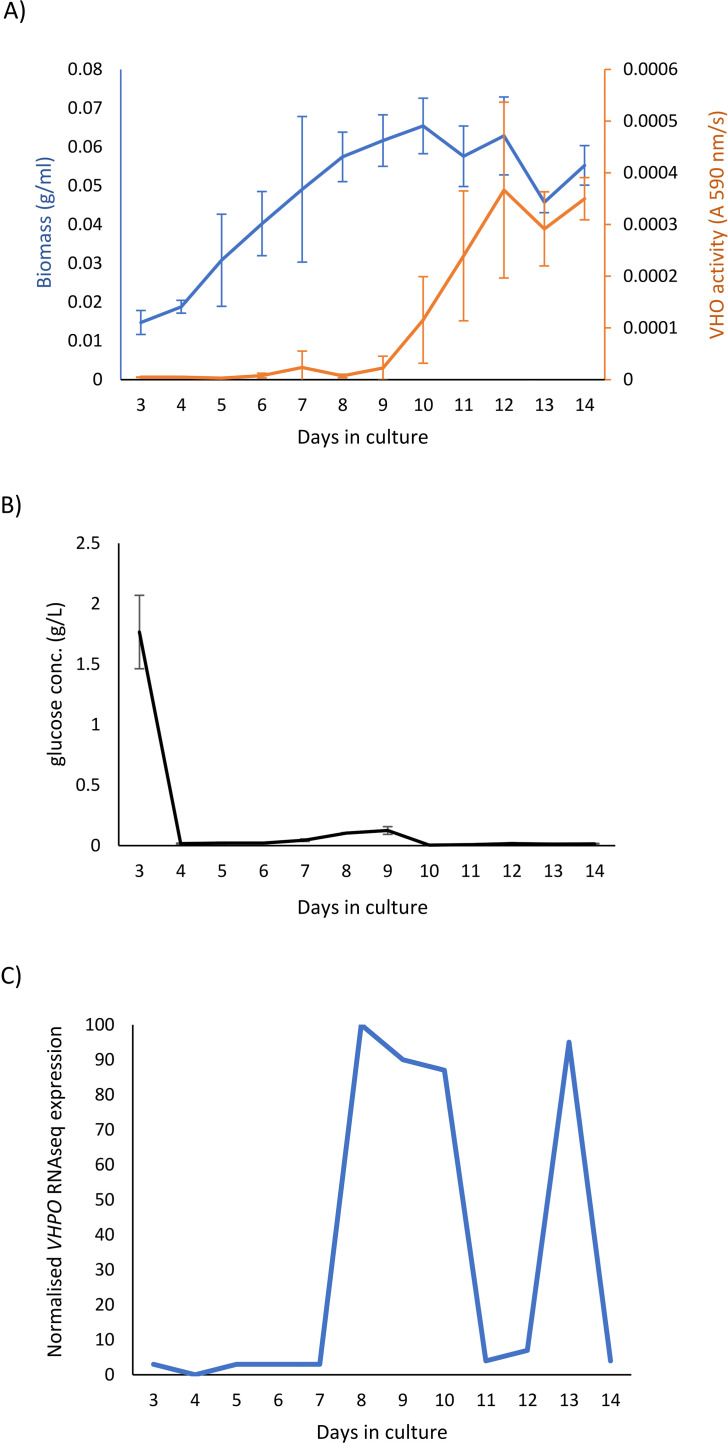
VHPO activity is induced in the culture idiophase. Culture kinetics of growth of *Curvularia* isolate 4388. (A) Triplicate cultures of fungal isolate 4388 were grown in LMM + 8 g/L of glucose and both sample mycelial biomass (blue line) and VHPO activity in culture supernatants (orange line) measured at days 3–14 inclusive. (B) Glucose concentrations were measured over 14 d in supernatant samples from the cultures of fungal isolate 4388 described in (A). (C) Relative *VHPO* transcript amounts present in RNAseq data derived from cultures described in (A). The maximum number of *VHPO* sequences per million reads (averaged from triplicate cultures) was designated 100 % and the remaining samples normalised relative to this value.

Triplicate 1 L cultures of fungal isolate 4388 were then grown in LMM + 8 g/L of glucose and 0.4 g/L agar and measured daily from days 3–14 inclusive for mycelial biomass, VHPO activity and glucose concentration in culture supernatant. Transcriptomics and metabolomics were undertaken on mycelia tissue from each time point. Biomass increased rapidly and plateaued at day 9 (Fig. 3a). Glucose was rapidly depleted from media and was undetectable by day 4 (Fig. 3b), although a small increase occurred on days 8 and 9 that may reflect storage compound remobilisation. VHPO activity was very low in culture supernatant until after day 9, plateauing at day 12 (Fig. 3a), suggesting enzyme upregulation occurs during culture idiophase [8,35].

**Fig. 4:**
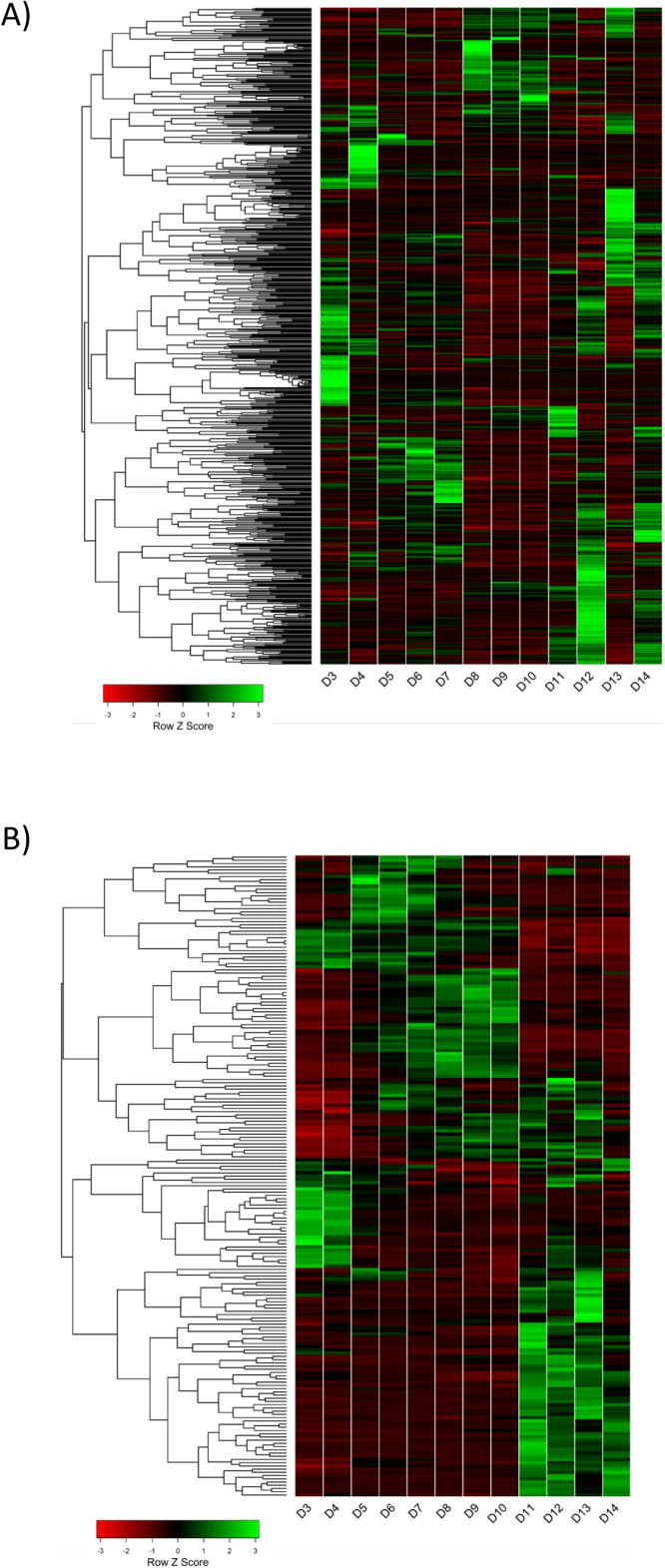
RNAseq and metabolomics analysis of a *Curvularia* isolate 4388 14-day culture. (A) An RNA expression heat map produced by hierarchical cluster analysis of 635 genes differentially expressed over the 3–14-day growth course of fungal isolate 4388. (B) Metabolite heatmap shown for the same culture of fungal isolate 4388 and generated by hierarchical cluster analysis. Expanded versions of these figures that include gene numbers and chemical compound ID numbers are shown in Supplementary Fig. S4 and S5. The numbers in these figures can be cross referenced in Supplementary Tables S3 and S5, respectively.

RNAseq transcriptomics analysis identified 11, 200 genes being expressed in these cultures (Supplementary Table S2) of which 635 were differentially expressed between at least two culture time points (*p* < 0.05) (Supplementary Table S3). Unique expression patterns were observed for each time point although days 3 + 4, 5 + 6 + 7 and 8 + 9 + 10 showed similarities (Fig. 4a), that were also observed in metabolite analyses (see below). VHPO transcript abundance in RNAseq data paralleled enzyme activity (Fig. 3a and c) consistent with transcriptional regulation of this gene [8]. Genes with an expression pattern similar to VHPO were enriched for secondary metabolism functions including amino acid, vitamin and alternative carbohydrate metabolism (Supplementary Table S4).

### 3.5 Metabolomics and toxogenomics

An essential requirement for livestock feed is an absence of toxicity. The genome of fungal isolate 4388 was analysed using the antiSMASH database to search for known microbial secondary metabolite biosynthetic gene clusters [36,37]. No strong indication of toxin producing gene clusters were identified with only a few genes showing limited homology to known toxin biosynthetic enzymes detected.

Large-scale polar metabolites analysis was undertaken on homogenised samples of fungal isolate 4388 cultures from the growth time course described. Approximately 200 metabolites were identified that were absent in control media samples (Supplementary Table S5) and none of which were known mycotoxins. Hierarchical cluster analysis showed metabolite profile similarities at days 3 + 4, 5 + 6 + 7, 8 + 9 + 10 and 11+12+13+14 (Fig. 4b). Similar clustering of RNAseq data occurred at these time points (Fig. 4a). Pathway analysis showed significant differences (*P* < 0.05) between sample time points for lysine degradation and aminoacyl-tRNA biosynthesis. These integrated transcriptomic and metabolomics analyses found no evidence of known toxin production throughout culture of fungal isolate 4388.

### 3.6 Optimised cultures of fungal isolate 4388 produce mM bromoform concentrations and inhibit methanogenesis *in vitro*

**Fig. 5:**
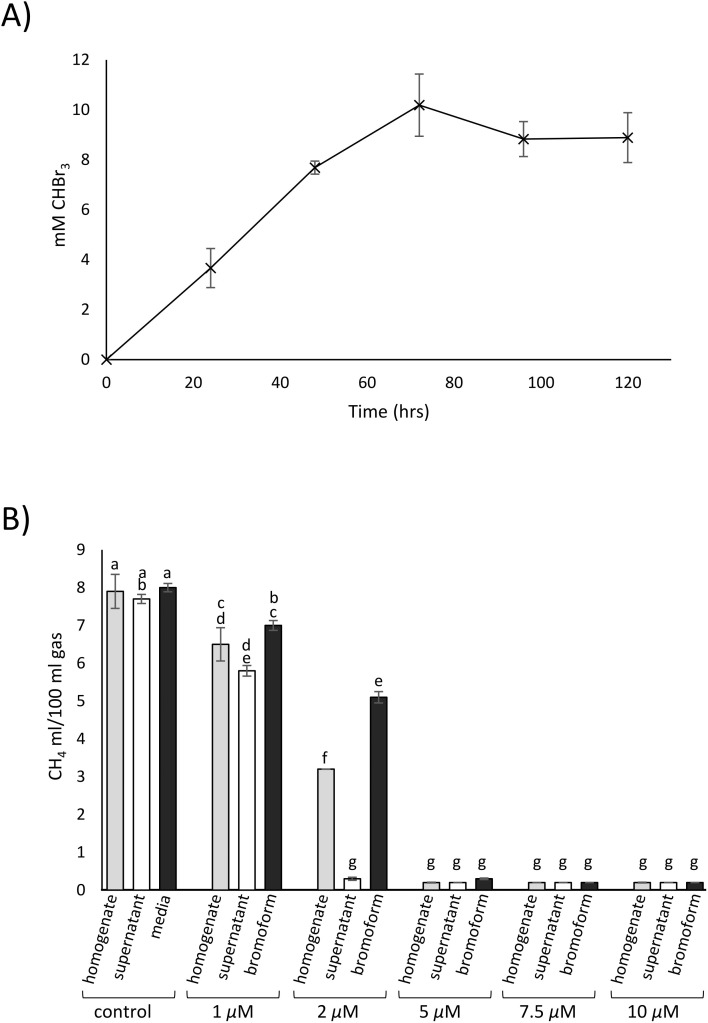
Bromoform producing cultures of fungal isolate 4388 inhibit bacterial methanogenesis. (A) Production of bromoform by *Curvularia* isolate 4388 cultures. Triplicate cultures were grown in LMM + 8 g/L glucose + 0.4 g/L agar prior to being supplemented daily with 100 mM H_2_O_2_ and 5 mM dimethyldimidone. Aliquots were taken at the time intervals indicated and bromoform concentration measured using GC-MS and quantified relative to a bromoform standard curve. (B) *In vitro* methane emissions from ovine rumen fluid samples after treatment with homogenate (grey columns) or supernatant (white columns) from bromoform producing cultures of fungal isolate 4388. Fungal extracts were added to 10 mL of rumen fluid to achieve final bromoform concentrations of 1–10 µM. Purified bromoform in liquid media was used for comparison (black columns). Control samples were homogenate and supernatant samples derived from non-bromoform producing cultures grown in the absence of 30 mM KBr (first two column at left) and media containing no bromoform (third column). Methane yields were determined over 24 h anaerobic incubation and expressed as mL CH_4_ per 100 mL of sample gas. Statistical analyses were undertaken using one way ANOVA and the Tukey-Kramer HSD post-hoc test. Columns with common letters above are not significantly different to each other. *P* < 0.05 was considered significant.

Bromoform synthesis by cultures of fungal isolate 4388 optimised for VHPO expression was investigated. Triplicate cultures were grown in LMM + 5 g/L of glucose and 0.4 g/L agar for two weeks at 28°C with shaking. Cultures were supplemented daily with 100 mM H_2_O_2_ and 5 mM dimethyldimidone. β-dicarbonyl compounds with low p*K*a values like dimethyldimidone are essential substrates for optimal VHPO-mediated bromoform production [27]. Maximum bromoform production occurred after 72 h treatment of the 14 day culture with up to 10 mM bromoform produced from some cultures (Fig. 5a).

Extracts from +KBr cultures of fungal isolate 4388 inhibited rumen fluid methanogenesis with similar efficacy to analytical bromoform when added at the same molarity (Fig. 5b). At bromoform concentrations of ≥5 µM, fungal extracts and analytical bromoform samples caused >95 % reduction in methane yield. Control samples of fungal isolate 4388 grown without KBr did not inhibit methanogenesis *in vitro*, indicating that bromoform and not other fungal products were the inhibitory agent (Fig. 5b). Total gas production was reduced by 3–4 % in samples with 1 and 2.5 µM bromoform (*P* < 0.05) but was similar to substrate control samples at higher bromoform concentrations (*P* > 0.05) (Supplementary Table S6).

*In vitro* fermentation with culture supernatant or homogenate samples did not result in significant changes in total volatile fatty acid (VFA) concentrations compared with the substrate control (Supplementary Table S6). The supernatant at doses ≥5 µM bromoform significantly decreased acetate (*P* < 0.05) relative to the substrate control, while the 7.5 and 10 µM samples compared with the 1 µM bromoform standard increased propionate (Supplementary Table S6). Butyrate concentrations increased with dose in all three treatments but was not consistently significant compared with 1 µM concentrations in homogenate, supernatant or bromoform standard. A change (*P* < 0.05) in acetate to propionate ratio (A:P) was observed with homogenate, supernatant and bromoform standard containing ≥2.5 *µ*M compared with 1 µM concentrations. The NH_3_ concentration was not significantly affected by any treatment and remained within a range of 17 to 27.9 mg/L.

## 4 Discussion

*Curvularia inaequalis* and *C. clavata* have been found in diverse habitats including soil, wood and on plants. The endogenous role of VHPO in *C. inaequalis* is suggested to be degradation of lignocellulose and penetration of plant cell walls [22,34]. This fungus has been shown to produce chlorinated lignin fragments when inoculated onto gymnosperm wood [23]. The probable carbon catabolite repression exerted upon this gene, observed here and in other studies [8,34], is consistent with the proposed secondary metabolic role of this enzyme.

The optimisation of *Curvularia* isolate 4388 cultures enabled bromoform production at concentrations up to 10 mM (= 2.53 g/L). These bromoform concentrations are theoretically sufficient to suppress methanogenesis when included as a minor (by weight) ingredient in ruminant diets. Feeding an alternative source of bromoform, namely *Asparagopsis taxiformis,* to cattle has been shown to significantly reduce enteric methane emissions [13]. To achieve almost complete mitigation *Asparagopsis* containing 6.55 mg of bromoform/g DM was fed at a rate of 0.2 % on an organic matter intake basis. This inclusion was equivalent to 120 mg of bromoform per animal per day or 250 µg of bromoform per kg live weight for the animals used in that trial [13]. To achieve similar dietary concentrations of bromoform, 47 mL of a *Curvularia* culture producing 10 mM bromoform would be required per animal per day. The *in vitro* data observed here for total VFA, acetate, propionate and butyrate concentration are similar to those reported after 72 h incubations using *Asparagopsis* and a Rhodes grass hay substrate [38]. In the current work *in vitro* fermentation of the substrate was not negatively affected by extracts of fungal isolate 4388. This was demonstrated by only minor effects on total gas production, no effects on total VFA and the reduction in ratio of acetate to propionate, all of which confirms the potential of this fungus as an alternative source of bromoform for mitigating enteric methanogenesis.

An alternative to biological sources of methanogenesis inhibitors like *Asparagopsis* and now *Curvularia*, are synthetic compounds like 3-nitrooxypropanol (3-NOP) which gives a reported mean reduction in enteric methane of 30 % [39]. The 3-NOP compound, now marketed as Bovaer®, structurally mimics methyl-coenzyme M reductase and competitively inhibits the final catalytic step of methanogenesis by ruminal archaea [7]. Whilst food products from naturally derived sources resonate positively with consumers, widespread adoption of methane abatement technologies across international markets will depend on efficacy, cost and regulatory approval. Compared with a 3-NOP dose of 100 mg/kg dry matter intake to achieve a considered optimal level of mitigation [39], a dietary bromoform inclusion of ∼13 mg/kg dry matter intake results in a similar outcome for reducing enteric methane from cattle [13].

Obvious questions associated with fungal derived supplements are potential product toxicity and acceptance by livestock when incorporated into the diet. Some *Curvularia* species are known to produce mammalian toxins including squalestatin S1 [40], methyl 5-(hydroxymethyl) furan-2-carboxylate [41], curvularol [42], curvularin [43] and cytochalasin A and B [44]. However, there are no reported mammalian toxins produced by *C. inaequalis*, or *C. clavata* to our knowledge; consistent with the absence of known toxin gene clusters in the genome sequence of fungal isolate 4388 and the absence of known toxins in its metabolomic analyses. In addition, 4388 samples grown in the absence of KBr and incapable of producing bromoform showed little inhibition of *M. smithii* growth and had no effect on methanogenesis or *in vitro* fermentation characteristics. Although these analyses did not include apparent digestibility, pH or hydrogen yield they can be assessed in future assays. *Curvularia* species have been reported as possible human allergens and rarely as opportunistic pathogens [45]. However, supernatant samples of fungal isolate 4388 contained minimal fungal tissue and therefore have reduced allergenicity potential but have similar *in vitro* methanogenesis suppression efficacy to homogenised cultures.

The use of bromoform as a feed supplement in ruminant production systems, regardless of the source, also raises environmental and consumer health concerns. The potential environmental impact of introducing bromoform into livestock systems based on the cultivation of *Asparagopsis* has been described [46]. In brief, the bromoform lost into the atmosphere from the production of a naturally derived halogenated methane analogue (HMA) to supply 50 % of feedlot and dairies in Australia would amount to <0.02 % of the global ozone depletion potential weighted emissions. Microbial fermentation offers greater bromoform containment possibilities during production due to the closed nature of fermentation platforms. In addition, bromoform is not formally listed as an ozone depleting substance in a Montreal Protocol context, because it is predominantly generated by natural sources [47] and is also regarded as a very short-lived substance having an atmospheric lifetime of less than six months.

Nevertheless, for bromoform to be accepted under regulatory guidelines as a feed additive for methane mitigation, it is essential to demonstrate that meat and edible offal from livestock fed this HMA is safe for human consumption and that elevated bromoform levels are not detected in tissues or meat products. As a reference point the World Health Organisation standard for bromoform in drinking water for human consumption is 100 μg/L. The LD50 of bromoform in rats is around 1200 mg of bromoform/kg/day and no effect on these animals was observed at concentrations of 500 mg/kg/day [48]. No bromoform residues have been found in samples of kidney, liver, fat, muscle tissue or milk taken from sheep [11] and beef cattle [13,14]. These results independently support recent *in vitro* studies demonstrating dehalogenation of CHBr_3_ to CH_2_Br_2_ by rumen microbes and then likely CH_3_Br, and finally methane and bromide [49]. The need for monitoring actual bromoform release in livestock environments appears unwarranted given the extent of rumen degradation and targeted mode of action, but *in vivo* investigations will be required to validate the suggested pathway for degradation of this HMA in the rumen.

## 5 Conclusion

The robust expression of VHPO in a culturable fungus creates an exciting new potential alternative for enteric methane mitigation from ruminant livestock. Being a culturable fungus amenable for industrial scale fermentation using very basic media ingredients has significant advantages over existing naturally occurring alternatives for producing bromoform. In addition, this fungal enzyme is highly stable in H_2_O_2,_ solvents and at higher temperatures [18,50,51] making further processing and product development opportunities possible.

## CRediT authorship contribution statement

**Thomas Loan:** Writing – review & editing, Validation, Methodology, Investigation. **Avinash Karpe:** Writing – review & editing, Investigation, Formal analysis. **Saeid Babaei:** Writing – review & editing, Formal analysis. **Stuart Denman:** Investigation, Formal analysis. **Chunghong Chen:** Formal analysis. **Matthias Joust:** Formal analysis. **Kristy Lam:** Methodology, Investigation. **Dawar Hussain:** Methodology, Investigation. **Sapna Pillai Vibhakaran:** Methodology, Investigation. **Matthew Callaghan:** Writing – review & editing, Investigation, Funding acquisition. **Abed Chaudhury:** Supervision, Conceptualization. **Karen Paco:** Methodology, Investigation. **Nigel Tomkins:** Writing – review & editing, Investigation, Funding acquisition. **Tristan Yang:** Methodology, Investigation. **Stephanie Payne:** Methodology, Investigation. **Michael Ayliffe:** Writing – review & editing, Supervision, Project administration, Funding acquisition, Formal analysis. **Ming Luo:** Writing – review & editing, Supervision, Project administration, Funding acquisition, Formal analysis.

## Declaration of competing interest

The authors declare that LoamBio Pty. Ltd. is investigating possible commercialisation of these outcomes.

## Data Availability

Data will be made available on request.

## Appendix Supplementary materials

Image, application 1

Image, application 2

Image, application 3

Image, application 4

Image, application 5

Image, application 6

Image, application 7

Image, application 8

Image, application 9

Image, application 10

Image, application 11
