# Supplementary material for: Biosynthesis of bromoform by Curvularia fungi provides a natural pathway to mitigate enteric methane emissions from ruminants
Source: Biotechnol Rep (Amst). 2025 Jan 14;45:e00876. doi: 10.1016/j.btre.2025.e00876 (PMC11791322; doi:10.1016/j.btre.2025.e00876)
Supplement: Supplementary file 1 [file mmc1.docx]

| **Supplemental Table 6. Mean (± sem) *in vitro* total gas production (ml), methane yield (ml/ g DMi^), calculated reduction (%) in methane yield, total volatile fatty acids (VFA), acetate, propionate and butyrate (mMol/L), acetate: propionate ratio and ammonia concentration (mg/L) for substrate only, homogenate and supernatant controls and homogenate, supernatant and bromoform standards using ovine rumen fluid and an oaten chaff substrate incubated over 24 h.** | | | | | | | | | |
| --- | --- | --- | --- | --- | --- | --- | --- | --- | --- |
| **Treatment** | **Total gas (mL)** | **Methane yield** | | **Total VFA** | **Acetate** | **Propionate** | **Butyrate** | **A:P** | **NH_3_**  **mg/L** |
|  |  | **(mL/g DMi)** | **%* reduction** | **mMol/L** | | | |  |  |
|  |  |  |  |  |  |  |  |  |  |
| Control substrate only | 33.2^ab^ ± 0.08 | 26.7^a^ ± 0.41 | 0 | 85.5 ± 1.39 | 56.8^a^ ± 1.13 | 19.7^bc^ ± 0.20 | 7.3^ab^ ± 0.06 | 2.9^a^ ± 0.03 | 24.6 ± 1.59 |
|  |  |  |  |  |  |  |  |  |  |
| Control Homogenate | 33.1^abc^ ± 0.19 | 25.5^ab^ ± 0.08 | 4 | 84.7 ± 0.66 | 56.1^ab^ ± 0.57 | 19.7^bc^ ± 0.07 | 7.2^ab^ ± 0.04 | 2.8^ab^ ± 0.02 | 25.8 ± 1.32 |
| Control Supernatant | 33.3^a^ ± 0.37 | 26.0^a^ ± 1.75 | 3 | 82.9 ± 4.46 | 55.1^ab^ ± 3.10 | 19.2^c^ ± 1.01 | 6.9^b^ ± 0.29 | 2.9^a^ ± 0.01 | 23.8 ± 2.75 |
|  |  |  |  |  |  |  |  |  |  |
| Homogenate 1 µM | 32.0^e^ ± 0.14 | 20.9^cd^ ± 1.62 | 22 | 80.8 ± 0.76 | 52.3^abcd^ ± 0.70 | 19.8^bc^ ± 0.02 | 7.0^b^ ± 0.08 | 2.6^cd^ ± 0.03 | 24.3 ± 1.16 |
| Homogenate 2.5 µM | 31.9^e^ ± 0.18 | 10.3^f^ ± 0.03 | 62 | 79.0 ± 2.85 | 48.8^abcd^ ± 2.05 | 21.3^abc^ ± 0.55 | 7.3^ab^ ± 0.19 | 2.3^f^ ± 0.04 | 20.3 ± 0.62 |
| Homogenate 5 µM | 32.9^abcd^ ± 0.18 | 0.6^g^ ± 0.02 | 98 | 78.9 ± 2.08 | 47.5^abcd^ ± 1.26 | 22.2^abc^ ± 0.58 | 7.6^ab^ ± 0.22 | 2.1^gh^ ± 0.01 | 17.0 ± 0.25 |
| Homogenate 7.5 µM | 33.3^ab^ ± 0.03 | 0.6^g^ ± 0.01 | 98 | 81.9 ± 1.85 | 49.8^abcd^ ± 1.20 | 22.3^abc^ ± 0.46 | 8.2^ab^ ± 0.15 | 2.2^fg^ ± 0.01 | 17.7 ± 0.28 |
| Homogenate 10 µM | 32.5^bcde^ ± 0.15 | 0.5^g^ ± 0.03 | 98 | 78.9 ± 0.21 | 48.0^abcd^ ± 0.37 | 21.1^abc^ ± 0.27 | 8.1^ab^ ± 0.04 | 2.3^f^ ± 0.05 | 24.6 ± 1.66 |
|  |  |  |  |  |  |  |  |  |  |
| Supernatant 1 µM | 32.2^de^ ± 0.03 | 18.8^de^ ± 0.47 | 30 | 83.4 ± 2.99 | 53.4^abcd^ ± 1.99 | 21.1^abc^ ± 0.70 | 7.2^ab^ ± 0.22 | 2.5^de^ ± 0.01 | 22.9 ± 0.97 |
| Supernatant 2.5 µM | 33.1^abc^ ± 0.04 | 1.1^g^ ± 0.14 | 96 | 81.8 ± 1.65 | 49.4^abcd^ ± 1.13 | 23.1^abc^ ± 0.38 | 7.7^ab^ ± 0.13 | 2.1^gh^ ± 0.01 | 19.7 ± 1.13 |
| Supernatant 5 µM | 33.5^a^ ± 0.07 | 0.6^g^ ± 0.01 | 98 | 75.4 ± 2.53 | 45.2^cd^ ± 1.51 | 21.2^abc^ ± 0.71 | 7.5^ab^ ± 0.26 | 2.1^gh^ ± 0.01 | 22.0 ± 3.34 |
| Supernatant 7.5 µM | 33.6^a^ ± 0.07 | 0.6^g^ ± 0.00 | 98 | 74.8 ± 2.60 | 44.4d ± 1.64 | 20.8^abc^ ± 0.64 | 8.0^ab^ ± 0.28 | 2.1^gh^ ± 0.02 | 18.2 ± 0.32 |
| Supernatant 10 µM | 33.5^a^ ± 0.04 | 0.6^g^ ± 0.00 | 98 | 77.5 ± 1.76 | 46.6^bcd^ ± 1.31 | 20.8^abc^ ± 0.33 | 8.4^a^ ± 0.12 | 2.2^fg^ ± 0.03 | 27.9 ± 5.68 |
|  |  |  |  |  |  |  |  |  |  |
| Bromoform 1 µM | 32.4^cde^ ± 0.19 | 22.7^bc^ ± 0.58 | 15 | 81.7 ± 1.13 | 53.5^abcd^ ± 0.74 | 19.6^c^ ± 0.30 | 7.0^b^ ± 0.07 | 2.7^bc^ ± 0.01 | 20.6 ± 1.38 |
| Bromoform 2.5 µM | 32.1^e^ ± 0.08 | 16.5^e^ ± 0.49 | 38 | 85.2 ± 6.18 | 54.1^abc^ ± 3.88 | 21.9^abc^ ± 1.58 | 7.5^ab^ ± 0.57 | 2.5^e^ ± 0.00 | 19.4 ± 1.05 |
| Bromoform 5 µM | 33.1^abc^ ± 0.10 | 1.0^g^ ± 0.06 | 96 | 79.6 ± 4.54 | 48.1^abcd^ ± 2.83 | 22.7^abc^ ± 1.24 | 7.3^ab^ ± 0.41 | 2.1^gh^ ± 0.01 | 19.0 ± 2.38 |
| Bromoform 7.5 µM | 33.3^a^ ± 0.09 | 0.6^g^ ± 0.00 | 98 | 81.9 ± 1.22 | 49.0^abcd^ ± 1.06 | 23.5^a^ ± 0.19 | 7.8^ab^ ± 0.02 | 2.1^h^ ± 0.03 | 19.5 ± 0.95 |
| Bromoform 10 µM | 33.5^a^ ± 0.11 | 0.6^g^ ± 0.01 | 98 | 83.6 ± 0.50 | 50.0^abcd^ ± 0.27 | 23.6^ab^ ± 0.20 | 8.1^ab^ ± 0.02 | 2.1^gh^ ± 0.01 | 17.7 ± 0.97 |
|  |  |  |  |  |  |  |  |  |  |
| ^DMi = dry matter incubated *in vitro*, *percentage reduction compared with control substrate (oaten chaff only)  Means within a column followed by different superscripts are significantly different (P < 0.05). | | | | | | | | | |
|  |  |  |  |  |  |  |  |  |  |
