# Supplementary material for: Biosynthesis of bromoform by Curvularia fungi provides a natural pathway to mitigate enteric methane emissions from ruminants
Source: Biotechnol Rep (Amst). 2025 Jan 14;45:e00876. doi: 10.1016/j.btre.2025.e00876 (PMC11791322; doi:10.1016/j.btre.2025.e00876)
Supplement: Supplementary file 2 [file mmc2.pptx]

## Slide 1
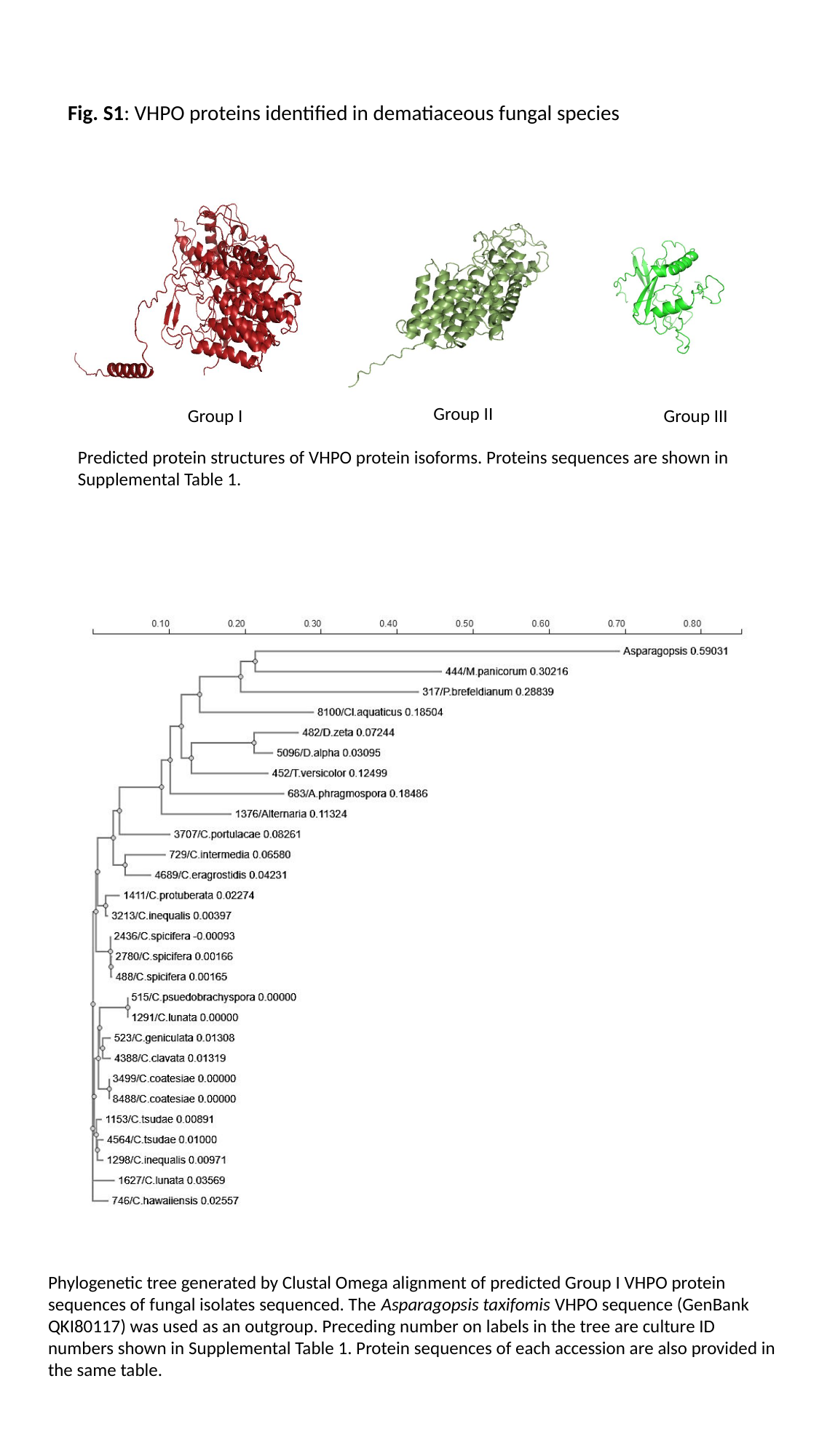

Fig. S1: VHPO proteins identified in dematiaceous fungal species
Group II
Group I
Group III
Predicted protein structures of VHPO protein isoforms. Proteins sequences are shown in Supplemental Table 1.
Phylogenetic tree generated by Clustal Omega alignment of predicted Group I VHPO protein sequences of fungal isolates sequenced. The Asparagopsis taxifomis VHPO sequence (GenBank QKI80117) was used as an outgroup. Preceding number on labels in the tree are culture ID numbers shown in Supplemental Table 1. Protein sequences of each accession are also provided in the same table.

## Slide 2
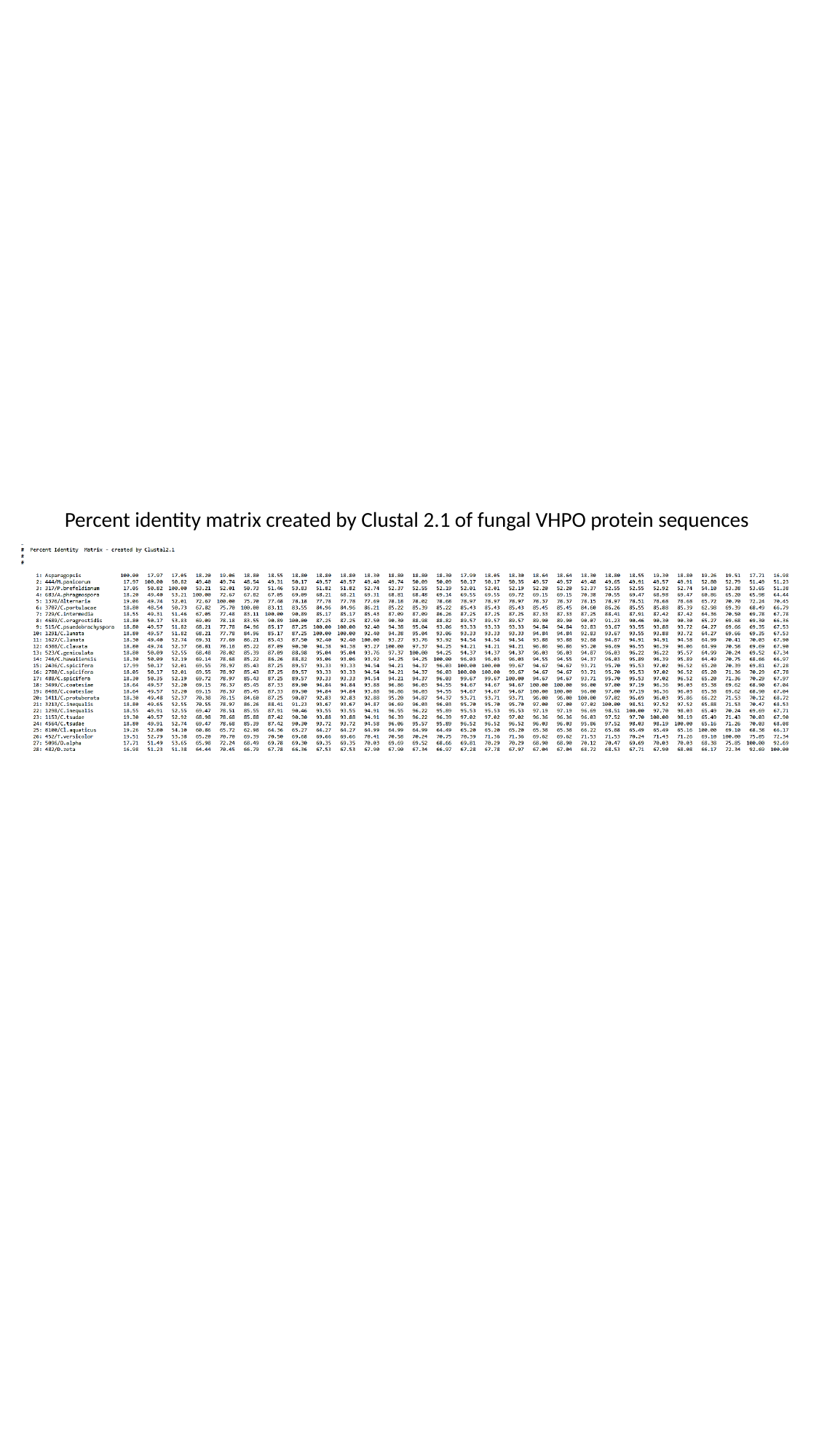

Percent identity matrix created by Clustal 2.1 of fungal VHPO protein sequences
