# Supplementary material for: Biosynthesis of bromoform by Curvularia fungi provides a natural pathway to mitigate enteric methane emissions from ruminants
Source: Biotechnol Rep (Amst). 2025 Jan 14;45:e00876. doi: 10.1016/j.btre.2025.e00876 (PMC11791322; doi:10.1016/j.btre.2025.e00876)
Supplement: Supplementary file 3 [file mmc3.pptx]

## Slide 1
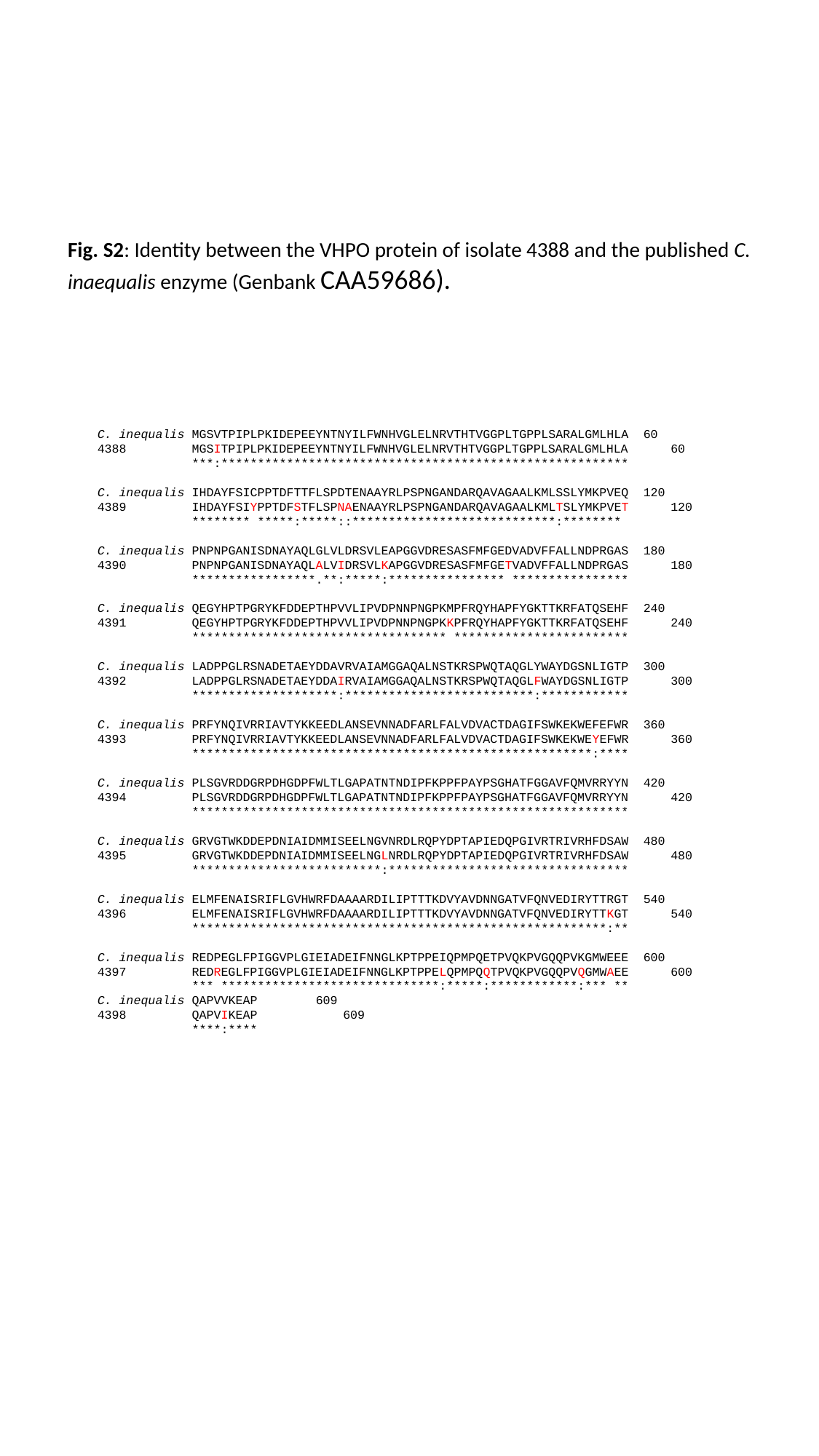

Fig. S2: Identity between the VHPO protein of isolate 4388 and the published C. inaequalis enzyme (Genbank CAA59686).
C. inequalis MGSVTPIPLPKIDEPEEYNTNYILFWNHVGLELNRVTHTVGGPLTGPPLSARALGMLHLA	60
 MGSITPIPLPKIDEPEEYNTNYILFWNHVGLELNRVTHTVGGPLTGPPLSARALGMLHLA	60
 ***:********************************************************
C. inequalis IHDAYFSICPPTDFTTFLSPDTENAAYRLPSPNGANDARQAVAGAALKMLSSLYMKPVEQ	120
 IHDAYFSIYPPTDFSTFLSPNAENAAYRLPSPNGANDARQAVAGAALKMLTSLYMKPVET	120
 ******** *****:*****::****************************:********
C. inequalis PNPNPGANISDNAYAQLGLVLDRSVLEAPGGVDRESASFMFGEDVADVFFALLNDPRGAS	180
 PNPNPGANISDNAYAQLALVIDRSVLKAPGGVDRESASFMFGETVADVFFALLNDPRGAS	180
 *****************.**:*****:**************** ****************
C. inequalis QEGYHPTPGRYKFDDEPTHPVVLIPVDPNNPNGPKMPFRQYHAPFYGKTTKRFATQSEHF	240
 QEGYHPTPGRYKFDDEPTHPVVLIPVDPNNPNGPKKPFRQYHAPFYGKTTKRFATQSEHF	240
 *********************************** ************************
C. inequalis LADPPGLRSNADETAEYDDAVRVAIAMGGAQALNSTKRSPWQTAQGLYWAYDGSNLIGTP	300
 LADPPGLRSNADETAEYDDAIRVAIAMGGAQALNSTKRSPWQTAQGLFWAYDGSNLIGTP	300
 ********************:**************************:************
C. inequalis PRFYNQIVRRIAVTYKKEEDLANSEVNNADFARLFALVDVACTDAGIFSWKEKWEFEFWR	360
 PRFYNQIVRRIAVTYKKEEDLANSEVNNADFARLFALVDVACTDAGIFSWKEKWEYEFWR	360
 *******************************************************:****
C. inequalis PLSGVRDDGRPDHGDPFWLTLGAPATNTNDIPFKPPFPAYPSGHATFGGAVFQMVRRYYN	420
 PLSGVRDDGRPDHGDPFWLTLGAPATNTNDIPFKPPFPAYPSGHATFGGAVFQMVRRYYN	420
 ************************************************************
C. inequalis GRVGTWKDDEPDNIAIDMMISEELNGVNRDLRQPYDPTAPIEDQPGIVRTRIVRHFDSAW	480
 GRVGTWKDDEPDNIAIDMMISEELNGLNRDLRQPYDPTAPIEDQPGIVRTRIVRHFDSAW	480
 **************************:*********************************
C. inequalis ELMFENAISRIFLGVHWRFDAAAARDILIPTTTKDVYAVDNNGATVFQNVEDIRYTTRGT	540
 ELMFENAISRIFLGVHWRFDAAAARDILIPTTTKDVYAVDNNGATVFQNVEDIRYTTKGT	540
 *********************************************************:**
C. inequalis REDPEGLFPIGGVPLGIEIADEIFNNGLKPTPPEIQPMPQETPVQKPVGQQPVKGMWEEE	600
 REDREGLFPIGGVPLGIEIADEIFNNGLKPTPPELQPMPQQTPVQKPVGQQPVQGMWAEE	600
 *** ******************************:*****:************:*** **
C. inequalis QAPVVKEAP	609
 QAPVIKEAP	609
 ****:****
