# Supplementary material for: Biosynthesis of bromoform by Curvularia fungi provides a natural pathway to mitigate enteric methane emissions from ruminants
Source: Biotechnol Rep (Amst). 2025 Jan 14;45:e00876. doi: 10.1016/j.btre.2025.e00876 (PMC11791322; doi:10.1016/j.btre.2025.e00876)
Supplement: Supplementary file 4 [file mmc4.pptx]

## Slide 1
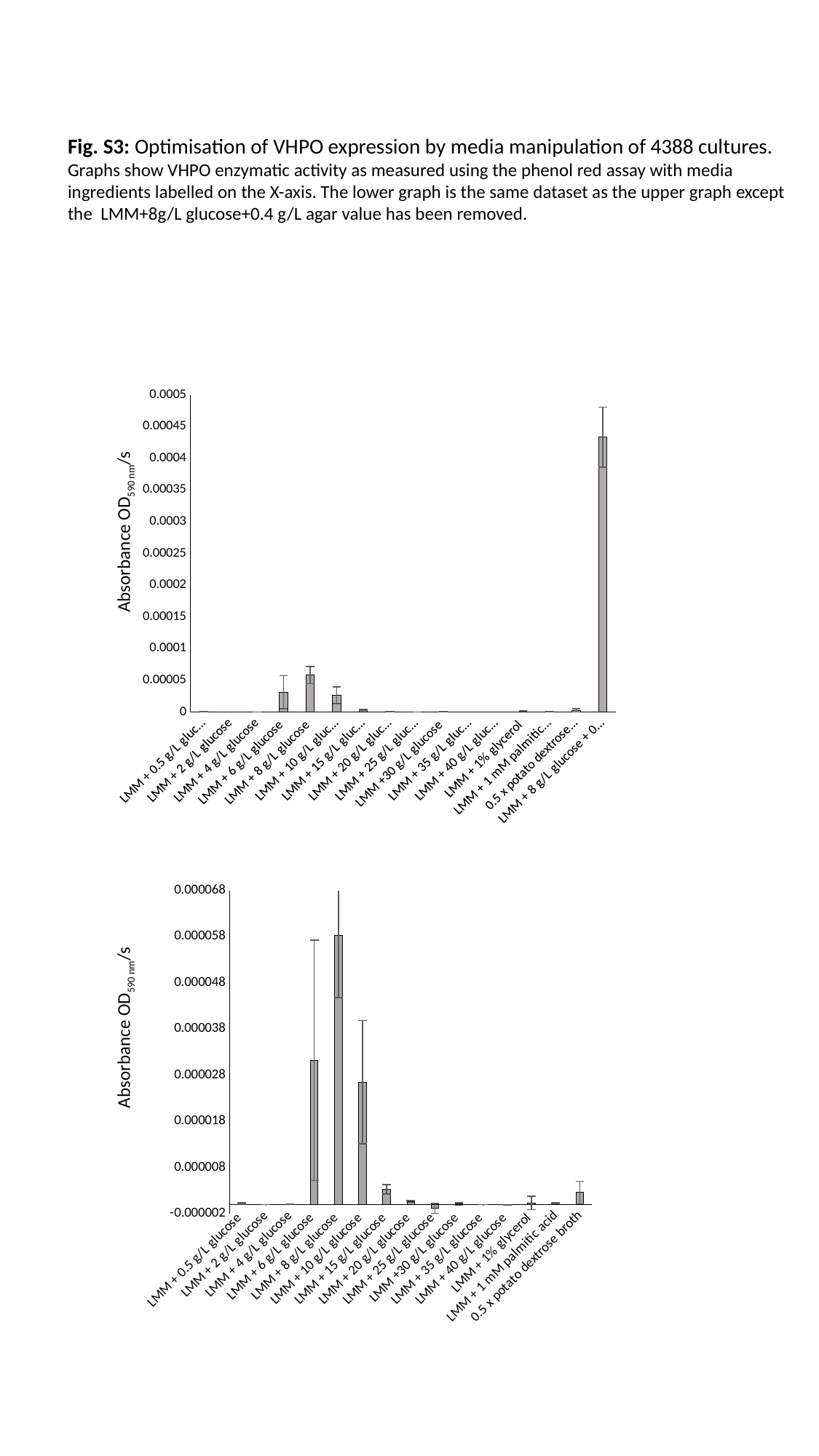

Fig. S3: Optimisation of VHPO expression by media manipulation of 4388 cultures.
Graphs show VHPO enzymatic activity as measured using the phenol red assay with media ingredients labelled on the X-axis. The lower graph is the same dataset as the upper graph except the LMM+8g/L glucose+0.4 g/L agar value has been removed.
### Chart
| Category | |
|---|---|
| LMM + 0.5 g/L glucose | 2.5737405882352936e-07 |
| LMM + 2 g/L glucose | -2.7705666666666667e-08 |
| LMM + 4 g/L glucose | 1.0555933333333333e-07 |
| LMM + 6 g/L glucose | 3.122873e-05 |
| LMM + 8 g/L glucose | 5.824933333333333e-05 |
| LMM + 10 g/L glucose | 2.6507333333333333e-05 |
| LMM + 15 g/L glucose | 3.3056666666666666e-06 |
| LMM + 20 g/L glucose | 6.677016666666666e-07 |
| LMM + 25 g/L glucose | -8.239976666666666e-07 |
| LMM +30 g/L glucose | 1.20673e-07 |
| LMM + 35 g/L glucose | -2.4321999999999998e-08 |
| LMM + 40 g/L glucose | -7.318133333333333e-08 |
| LMM + 1% glycerol | 3.3723633333333336e-07 |
| LMM + 1 mM palmitic acid | 2.573276666666667e-07 |
| 0.5 x potato dextrose broth | 2.6070219999999995e-06 |
| LMM + 8 g/L glucose + 0.4 g/L agar | 0.000433333333333333 |Absorbance OD590 nm/s
### Chart
| Category | |
|---|---|
| LMM + 0.5 g/L glucose | 2.5737405882352936e-07 |
| LMM + 2 g/L glucose | -2.7705666666666667e-08 |
| LMM + 4 g/L glucose | 1.0555933333333333e-07 |
| LMM + 6 g/L glucose | 3.122873e-05 |
| LMM + 8 g/L glucose | 5.824933333333333e-05 |
| LMM + 10 g/L glucose | 2.6507333333333333e-05 |
| LMM + 15 g/L glucose | 3.3056666666666666e-06 |
| LMM + 20 g/L glucose | 6.677016666666666e-07 |
| LMM + 25 g/L glucose | -8.239976666666666e-07 |
| LMM +30 g/L glucose | 1.20673e-07 |
| LMM + 35 g/L glucose | -2.4321999999999998e-08 |
| LMM + 40 g/L glucose | -7.318133333333333e-08 |
| LMM + 1% glycerol | 3.3723633333333336e-07 |
| LMM + 1 mM palmitic acid | 2.573276666666667e-07 |
| 0.5 x potato dextrose broth | 2.6070219999999995e-06 |Absorbance OD590 nm/s
