# Supplementary material for: Biosynthesis of bromoform by Curvularia fungi provides a natural pathway to mitigate enteric methane emissions from ruminants
Source: Biotechnol Rep (Amst). 2025 Jan 14;45:e00876. doi: 10.1016/j.btre.2025.e00876 (PMC11791322; doi:10.1016/j.btre.2025.e00876)
Supplement: Supplementary file 6 [file mmc6.pdf]

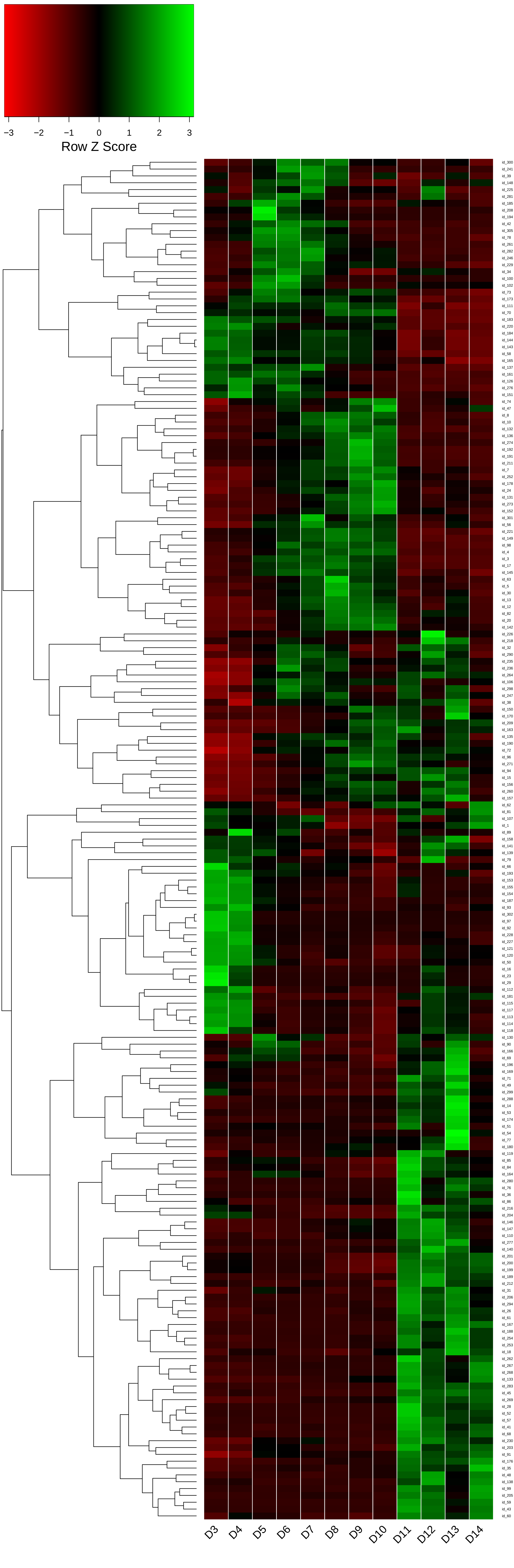

−3 −2 −1 0 1 2 3
Row Z Score
D3
D4
D5
D6
D7
D8
D9
D10
D11
D12
D13
D14
